# RBM15 Enhances 5-Fluorouracil Drug Sensitivity and Suppresses Gastric Cancer Progression by Modulating N6-Methyladenosine Modification of ECT2-Dependent IGF2BP3

**DOI:** 10.34133/research.1108

**Published:** 2026-02-02

**Authors:** Xingyu Zhu, Hao Chen, Kang Xu, Yuan Liu, Han Li, Yaodong Sang, Yulong Zhao, Xinyu Liu, Xiaohan Wang, Xiaoling Cui, Baoshan Cai, Liang Shang, Changqing Jing, Wei Chong, Leping Li

**Affiliations:** ^1^ Department of Gastrointestinal Surgery, Shandong Provincial Hospital Affiliated to Shandong First Medical University, Jinan 250021, China.; ^2^ Shandong Provincial Laboratory of Translational Medicine Engineering for Digestive Tumors, Shandong Provincial Hospital, Jinan 250021, China.; ^3^ Medical Science and Technology Innovation Center, Shandong First Medical University and Shandong Academy of Medical Sciences, Jinan 250021, China.; ^4^ Clinical Research Center of Shandong University, Clinical Epidemiology Unit, Qilu Hospital of Shandong University, Jinan 250021, China.; ^5^ Department of Epidemiology and Health Statistics, School of Public Health, Cheeloo College of Medicine, Shandong University, Jinan 250021, Shandong, China.; ^6^ Department of Gastroenterological Surgery, The First Affiliated Hospital of Shandong First Medical University, Jinan 250021, China.

## Abstract

Gastric cancer (GC) remains a leading cause of global cancer mortality. Analysis of clinical tissues and multiple cohorts (TCGA, ACRG, Singapore, KUGH) associated high RBM15 expression with favorable prognosis. Functional assays in vitro and in vivo demonstrated that RBM15 suppresses GC cell proliferation, migration, and invasion. Integrated RNA-seq and bioinformatics analyses identified the oncogene ECT2 and the epithelial–mesenchymal transition (EMT) pathway as key downstream effectors of RBM15. Mechanistically, RBM15 regulates the m6A methylation of ECT2 mRNA at the 2,909-base pair site, which modulates its binding to the reader protein IGF2BP3, as confirmed by MeRIP, RIP-qPCR, and RNA pull-down assays. A luciferase reporter assay further validated that this m6A modification regulates ECT2 expression. Furthermore, animal and patient-derived organoid models revealed that RBM15 enhances the sensitivity of GC to 5-fluorouracil (5-FU) chemotherapy in an ECT2-dependent manner. In conclusion, this study defines a novel RBM15/IGF2BP3–ECT2 signaling axis that regulates EMT and chemosensitivity in GC via m6A methylation, providing both mechanistic insights and a potential therapeutic strategy.

## Introduction

As a globally prevalent gastrointestinal cancer exceeding 1 million annual diagnoses, gastric cancer (GC) simultaneously emerges as the world’s third most lethal oncologic disease [1,2]. As a key prognostic indicator, the clinical staging of GC at diagnosis exerts primary influence on therapeutic response and overall survival. Without distinct symptomatic presentation in initial GC phases [2,3], differentiation from common gastrointestinal disorders is rarely straightforward, making molecular biomarker discovery for early screening an immediate priority [4–6]. The advent of molecular subtyping of GC has provided novel perspectives for screening genes related to early-stage GC progression and prognosis [7]. Recently, molecular targeted drugs and immunotherapy have made tremendous progress, such as tyrosinase inhibitors and immune checkpoint inhibitors, which greatly improved the prognosis of patients [8,9]. By comparing the disparities in gene expression among different molecular subtypes, some genes that are closely associated with specific molecular subtypes can be identified. Functioning as molecular orchestrators, these genetic elements potentially dictate the trajectory of malignant disease—from initial transformation through metastatic competence [10,11]. Additionally, through long-term follow-up and analysis of the prognoses of patients with different molecular subtypes, gene markers closely linked to prognosis can be screened out, offering robust evidence for clinical physicians to predict patient prognoses and formulate personalized treatment regimens. Meanwhile, intrinsic or acquired resistance to chemotherapy frequently leads to poor or ineffective treatment responses, posing a marked challenge in the management of GC [12]. Currently available biomarkers for early GC detection, such as carcinoembryonic antigen (CEA), α-fetoprotein (AFP), carbohydrate antigen 19-9 (CA19-9), CA72-4, and CA125 [13–17], lack adequate sensitivity and specificity. The demonstrated limitations in current practice necessitate emergent development of novel biomarkers, crucial for achieving dual breakthroughs in early GC interception and chemotherapy response optimization. As a pivotal epigenetic regulator, m6A modification pervasively modulates the RNA lifecycle continuum—from transcriptional initiation through spatial conformation folding to eventual degradation—serving as the central processing unit of RNA metabolism [18]. The m6A methyltransferase complex (integrating METTL3, METTL14, WTAP, METTL16, VIRMA, and RBM15) has been extensively characterized. As its paramount scaffolding subunit, VIRMA orchestrates spatial targeting of m6A modifications to 3′ untranslated regions (UTRs) and termination codon-adjacent genomic segments [19,20]. The erasers, mainly consisting of FTO and ALKBH5, mediate the reversible demethylation of m6A. For example, FTO is implicated in fat metabolism and energy homeostasis [21]. As a master coordinator of RNA nuclear export, metabolic flux, and expression networks, ALKBH5 exhibits testis-dominant expression with ancillary cardiac and cerebral presence [22]. The most well-characterized m6A “readers” are the YTHDF (YTH domain family) and IGF2BP (insulin-like growth factor 2 mRNA-binding protein 3) families [23]. Despite the substantial research on various “writers”, the role of RBM15 remains relatively under-explored. While RBM15 exhibits oncogenic acceleration in laryngeal squamous carcinoma [24], and tumor-suppressive constraint upon depletion in colorectal cancer [25], its pathomechanistic footprint in gastric oncogenesis constitutes a critical research void awaiting illumination.

IGF2BP3, as an RNA-binding protein, plays a dual role in embryonic development and tumorigenesis by regulating the stability of key mRNAs. Its ​​oncofetal protein characteristics​​ make it a highly promising cancer diagnostic biomarker and therapeutic target, with particularly significant value in overcoming targeted therapy resistance [26]. Under pathological conditions, IGF2BP3 is abnormally overexpressed in multiple cancers [26]. In cervical cancer, high IGF2BP3 expression is closely associated with advanced cancer stage, lymph node metastasis, and poor patient prognosis [27]. In colorectal cancer, IGF2BP3 stabilizes epidermal growth factor receptor (EGFR) mRNA in an ​​m6A-dependent manner​​, promoting tumor progression and conferring resistance to cetuximab [28]. The oncogenic mechanisms of IGF2BP3 primarily include promoting cell proliferation​​ (by regulating cell cycle-related genes) [27], inhibiting apoptosis​​ (by suppressing proapoptotic genes), facilitating tumor invasion and metastasis​​ [by modulating the expression and activity of proteases such as matrix metalloproteinases and enhancing lipid metabolism through SCD (stearoyl-coenzyme A desaturase) up-regulation] [29], and inducing therapy resistance​​ (e.g., cetuximab resistance via EGFR mRNA stabilization) [28,30].

Epithelial cell transformation sequence 2 (ECT2) functions as the canonical guanine nucleotide exchange factor catalyzing activation of Rho guanosine triphosphatases (GTPases)—specifically RhoA, Rac1, and Cdc42—through guanosine diphosphate (GDP)/guanosine triphosphate (GTP) cycling regulation [31]. ECT2 facilitates the exchange of GDP for GTP, thereby converting Rho GTPases from an inactive to an active state, which subsequently drives actin cytoskeleton remodeling [32]. ECT2 has been implicated in malignant transformation, tumorigenesis, and metastasis [33]. Its oncogenic effects are influenced by its subcellular localization. ECT2’s NLS (nuclear localization signal)-mediated nuclear predominance [34] contrasts with cytoplasmic cytokinesis functions [35], establishing compartment-specific oncogenic programs: Nuclear pools drive transformation (e.g., lung: Rac1→ribosome biogenesis [36]), while cytoplasmic retention in ovarian cancer activates Rac1–Pak–Mek–Erk proliferative cascades [37]. Hepatocellular carcinoma further reveals ECT2’s Rho–Elk-mediated triad of pro-growth, anti-apoptotic, and pro-metastatic signals [38]. The absence of GC correlates defines a critical research frontier.

We have deciphered a functional RBM15–IGF2BP3 m6A/ECT2 signaling axis that modulates the epithelial–mesenchymal transition (EMT)-driven malignant progression and chemoresponse in GC. This conclusion is robustly supported by multi-database survival analyses, which consistently indicate that high expression of RBM15 is associated with a better prognosis. Mechanistically, functioning as an m6A “writer” protein, RBM15 binds to the ECT2 transcript at a specific site [2,909 base pairs (bp)] and facilitates its m6A methylation. This modification is subsequently recognized and stabilized by the “reader” protein IGF2BP3, which directly binds to ECT2 mRNA, thereby enhancing its RNA stability and expression levels. The subsequent influence of ECT2 specifically regulates the EMT pathway, which in turn inhibits the proliferation, migration, and invasion of GC cells, as confirmed by both in vitro and in vivo functional assays. Furthermore, this axis impacts chemosensitivity in GC. Our experiments demonstrate that RBM15 enhances the sensitivity of GC cells and patient-derived organoids to 5-fluorouracil (5-FU) chemotherapy via its regulation of ECT2. The effects depend on the specific molecular interactions within the RBM15–IGF2BP3–ECT2 axis. Therefore, targeting this signaling axis can provide a novel and promising new therapeutic model for this subset of GC patient.

## Results

### RBM15 correlates with improved prognosis in GC

**Fig. 1.**
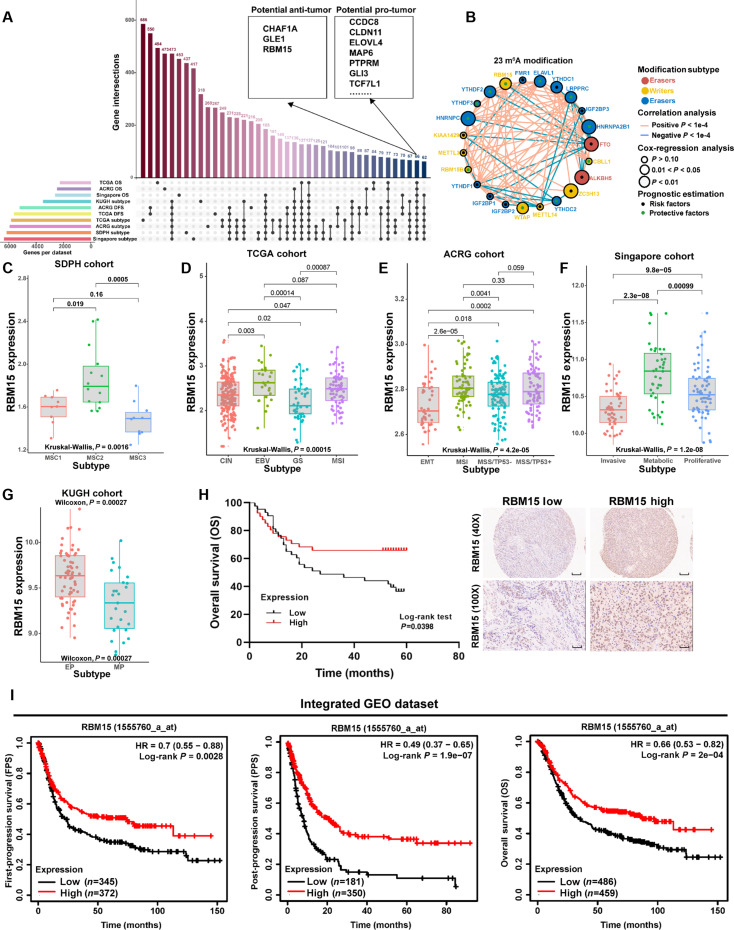
The survival analysis of RBM15 in human GC. (A) The analysis combined the TCGA, ACRG, Singapore, KUGH, and in-house SDPH cohorts. (B) Prognostic significance of these 23 m6A regulatory molecules. (C to G) Scatterplots showing RBM15 expression across molecular subtypes in the SDPH, TCGA, ACRG, Singapore, and KUGH cohorts, respectively. (H) Survival analysis among 84 GC patients based on RBM15 expression. The tissues comprised 84 GC tissue samples obtained from tissue microarrays provided by Shanghai Outdo Biotech Company (Shanghai, China) between January 2011 and December 2012 (40× scale bar = 200 μm, 100× scale bar = 100 μm). (I) Integrated analysis of the relationship between RBM15 expression and prognosis [first-progression survival (FPS), post-progression survival (PPS), and overall survival (OS)] in GC patients across the GC chip datasets GSE15459 (Singapore), GSE14210, GSE22377, GSE29272, GSE51105, and GSE62254.

Previous research has discerned several molecular subtypes of GC, each associated with unique molecular characteristics and clinical significance. To identify biomarkers associated with GC progression and prognosis, we screened for the intersection of differentially expressed genes (DEGs) across various molecular subtype and prognosis-related genes using data from The Cancer Genome Atlas (TCGA), Asian Cancer Research Group (ACRG), Singapore, KUGH (Korea University Guro Hospital), and in-house SDPH (Shandong Provincial Hospital) databases (Fig. 1A). Ultimately, 2 groups of gene markers were identified. Genes such as RBM15, CHAF1A, and GLE1 had potential antitumor roles, while genes like CCDC8, CLDN11, and ELOVL4 had protumor roles (Fig. 1A and Table S1).

Following our demonstration of 23-m6A-regulators’ therapeutic utility in colon cancer [39], RBM15 emerged as a GC biomarker. Clinically, elevated RBM15 conferred survival advantage (Fig. 1B) and coordinated with FTO/WTAP/IGF2BP3 expression axes (Fig. S1A and B). Mutational landscapes showed RBM15 alterations in 3% GC cases (third among m6A factors; Fig. S1C), while co-mutation analysis revealed selective partnerships with ELAVL1/IGF2BP2/YTHDC1/YTHDF1-2 (Fig. S1D).

RBM15 demonstrated expression heterogeneity. In the in-house SDPH cohort, RBM15 expression was significantly decreased in the MSC3 subtype related to tumor progression (Fig. 1C and Fig. S1E). In the TCGA cohort, RBM15 was highly expressed in the EBV (Epstein–Barr virus) and MSI (microsatellite instability) subtypes, both of which are associated with favorable prognosis (Fig. 1D and Fig. S1F). Similarly, in the ACRG cohort, RBM15 expression was elevated in the MSI and MSS (microsatellite stability)/TP53^+^ subtypes (Fig. 1E and Fig. S1G). In the Singapore cohort, the expression level of RBM15 in the invasive group was the lowest (Fig. 1F and Fig. S1H). In the KUGH cohort, the expression level of RBM15 in the epithelial phenotype (EP) group was higher than that in the mesenchymal phenotype (MP) group (Fig. 1G and Fig. S1I). A tissue microarray analysis was further conducted in 84 GC patients, and it was found that those in the RBM15 high-expression subgroup had a better prognosis (Fig. 1H and Table S2). Additionally, through Kaplan–Meier survival analysis of the TCGA cohort and integration of microarray data from multiple Gene Expression Omnibus (GEO) databases (including ACRG, Singapore, GSE15459, GSE14210, GSE22377, GSE29272, GSE51105, and GSE62254), it was further validated that high RBM15 expression was associated with prolonged survival in GC patients (Fig. 1I and Fig. S1J).

### RBM15 inhibited the proliferation, migration, and invasion of GC cells

**Fig. 2.**
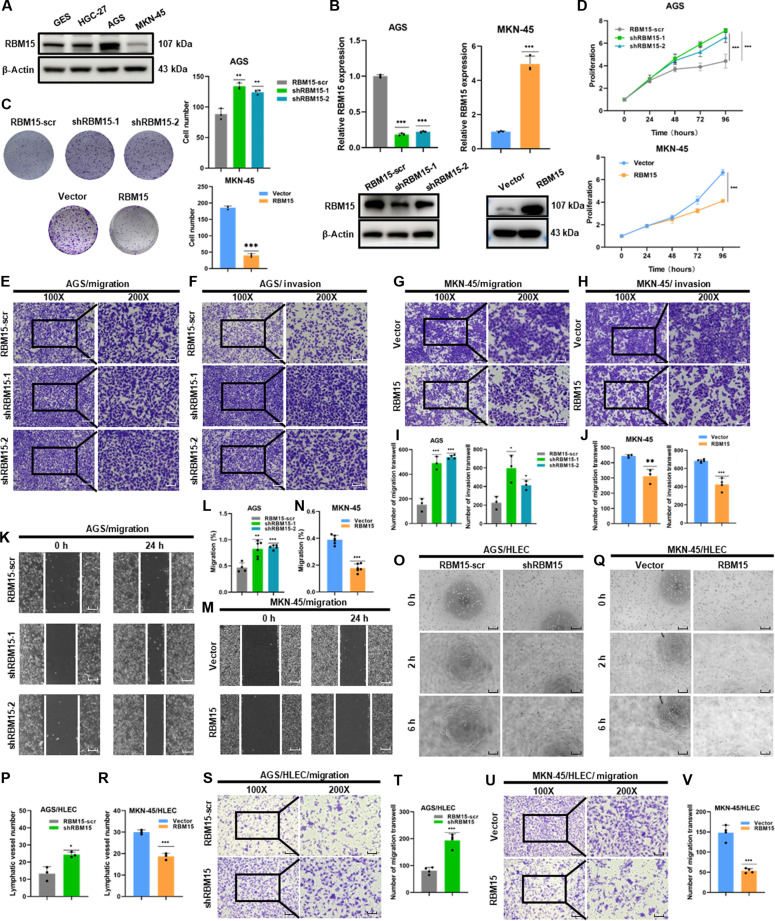
Influence of RBM15 knockdown or overexpression in GC cells. (A) Endogenous RBM15 expression in GC cell lines detected by Western blotting. (B) Knockdown efficiency in AGS cells and overexpression efficiency in MKN-45 cells confirmed by qPCR and Western blotting. (C and D) Cell proliferation assessed by colony formation and CCK-8 assays in AGS cells transfected with scramble control (scr) or 2 distinct shRNAs targeting RBM15 (shRBM15-1 and shRBM15-2), and in MKN-45 cells transfected with empty vector (Vector) or RBM15 overexpression plasmid (RBM15). (E, F, and I) Cell migration and invasion assays in AGS cells with RBM15-scr, shRBM15-1, and shRBM15-2 (RBM15 knockdown) (100× scale bar = 100 μm, 200× scale bar = 50 μm). (G, H, and J) Cell migration and invasion assays in MKN-45 cells with Vector (vector control) or RBM15 (RBM15 overexpression) (100×: scale bar = 100 μm, 200× scale bar = 50 μm). (K and L) Cell migration assay in AGS cells with RBM15-scr, shRBM15-1, and shRBM15-2 (RBM15 knockdown) (scale bar = 100 μm). (M and N) Cell migration assay in MKN-45 cells with Vector (vector control) or RBM15 (RBM15 overexpression) (scale bar = 100 μm). (O and P) Lymphatic vessel formation ability of HLEC cells cocultured with AGS cells with RBM15-scr, shRBM15-1, and shRBM15-2 (RBM15 knockdown) (scale bar = 100 μm). (Q and R) Lymphatic vessel formation ability of HLEC cells cocultured with MKN-45 cells with Vector (vector control) or RBM15 (RBM15 overexpression) (scale bar = 100 μm). (S and T) Migration of HLEC cocultured with AGS cells with RBM15-scr, shRBM15-1, and shRBM15-2 (RBM15 knockdown) by transwell assay (100× scale bar = 100 μm, 200× scale bar = 50 μm). (U and V) Migration of HLEC cocultured with MKN-45 cells with Vector (vector control) or RBM15 (RBM15 overexpression) (100× scale bar = 100 μm, 200× scale bar = 50 μm).

To validate the previous analysis from the perspective of cellular functions, a series of experiments were carried out to elucidate its molecular mechanisms at the cellular level. Based on the expression level of RBM15 in GC cells (Fig. 2A), we selected AGS cells for knockdown and MKN-45 cells for overexpression. AGS cells were transfected with short hairpin RNA (shRNA) to knock down RBM15 (shRBM15), and MKN-45 cells were engineered to overexpress RBM15 (Fig. 2B). In the colony formation assay (Fig. 2C) and cell counting kit-8 (CCK-8) assay (Fig. 2D), knockdown of RBM15 in AGS cells led to a significant increase in cell proliferation, while RBM15 overexpression in MKN-45 cells resulted in a marked decrease in proliferation. Furthermore, transwell assays (Fig. 2E, F, and I) and wound healing assays (Fig. 2K and L) demonstrated that RBM15 knockdown significantly enhanced cell migration and invasion, while RBM15 overexpression suppressed these behaviors (Fig. 2G, H, J, M, and N). Coculture experiments with human lymphatic endothelial cells (HLECs) further revealed that RBM15 knockdown increased the lymphangiogenic potential of HLECs (Fig. 2O and P), while RBM15 overexpression diminished this capability (Fig. 2Q and R). Additionally, RBM15 knockdown promoted the migratory ability of HLECs (Fig. 2S and T), while its overexpression inhibited HLEC migration (Fig. 2U and V).

Synthesizing experimental evidence, RBM15 is functionally validated as a gastric tumor suppressor that mechanistically constrains in vitro oncogenic processes: proliferative expansion, migratory activity, and invasive progression.

### RBM15 regulated EMT pathways

**Fig. 3.**
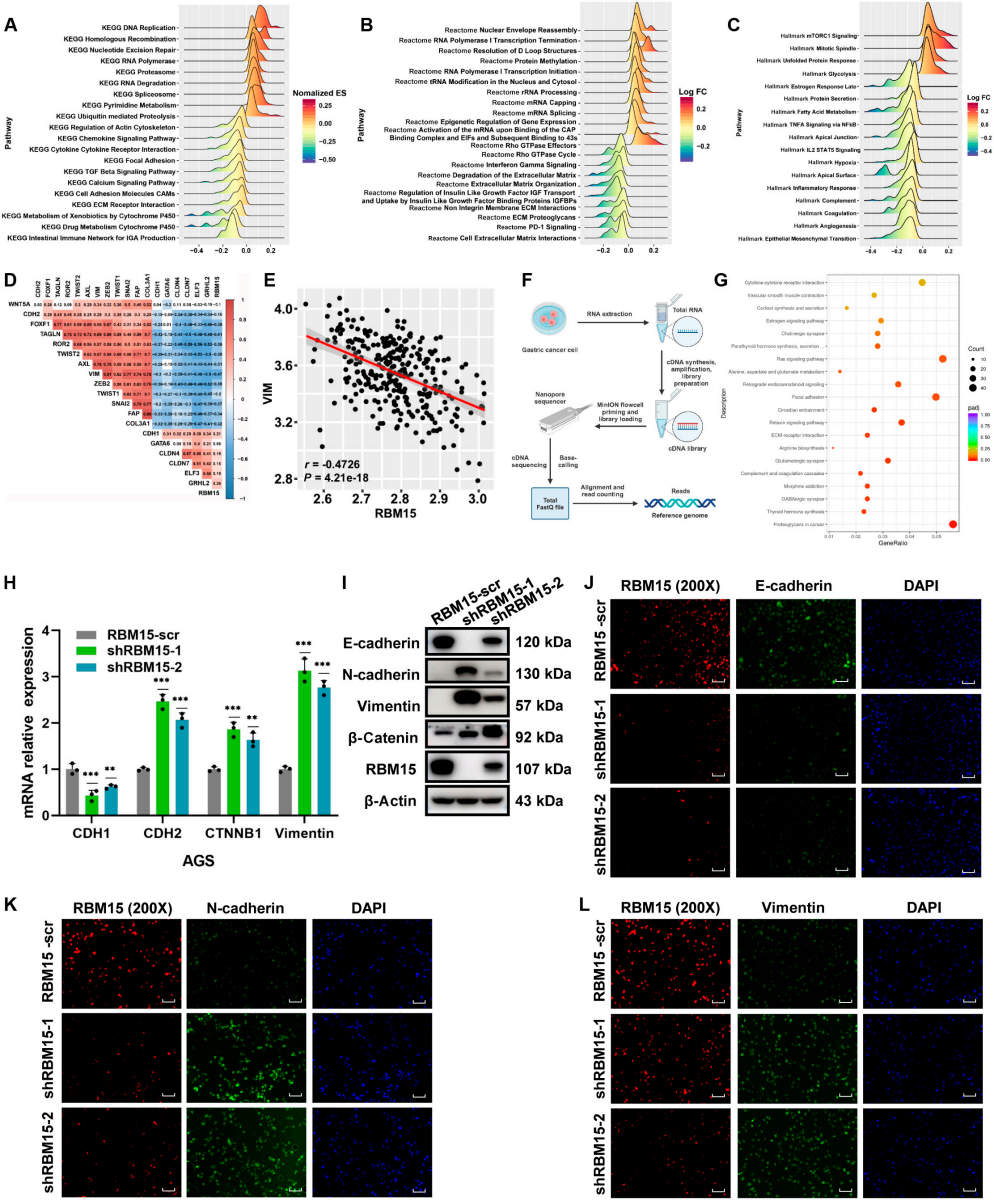
RBM15 regulated the EMT pathways. (A to C) Gene Set Enrichment Analysis (GSEA) of the ACRG cohort based on KEGG, Reactome, and Hallmark datasets. (D) Correlation between RBM15 and an EMT-related gene signature analyzed by Spearman’s rank test. (E) Correlations between RBM15 and VIM using Spearman analysis. (F) Flowchart of RNA sequencing. (G) KEGG pathway analysis of DEGs in RBM15-knockdown AGS cells from RNA-seq. (H and I) mRNA and protein expression levels of CDH1, CDH2, CTNNB1, and VIM in RBM15-knockdown AGS cells, determined by qPCR and Western blotting, respectively. (J to L) Immunofluorometric assay in AGS cells with shRBM15 about E-cadherin, N-cadherin, and Vimentin (200× scale bar = 50 μm).

To decode the pathway-level consequences of RBM15 dysregulation, subgroup-stratified Gene Set Enrichment Analysis (GSEA) (high versus low expression) was performed in the ACRG cohort. Integrating Kyoto Encyclopedia of Genes and Genomes (KEGG)/Reactome/Hallmark pathway databases, GSEA linked RBM15 to (a) m6A methylation-dependent activities including RNA processing and splicing, and (b) regulatory circuits of EMT, cellular adhesion, and transforming growth factor-β (TGF-β) signaling (Fig. 3A to C). Molecular subtype analysis further demonstrated that RBM15 expression was down-regulated in the EMT subtype (Fig. 1E and Fig. S1G). In GC, RBM15 demonstrated significant anticorrelation with mesenchymal markers [CDH2 (N-cadherin), VIM, ZEB2, TWIST1/2, SNAI2] while covarying positively with the epithelial marker CDH1 (E-cadherin) (Fig. 3D and E and Fig. S2A to F).

To further elucidate the mechanism, RNA-seq was performed on GC cells with RBM15 knockdown (Fig. 3F). The GSEA results reinforced that RBM15 is involved in pathways related to focal adhesion, extracellular matrix (ECM)–receptor interaction, etc. (Fig. 3G). The quantitative polymerase chain reaction (qPCR) quantification confirmed that RBM15 knockdown reciprocally regulated EMT markers: suppressing CDH1 mRNA while activating CDH2, CTNNB1, and VIM transcription (Fig. 3H). Conversely, RBM15 overexpression had the opposite effect (Fig. S2G). Western blot and immunofluorescence (IF) assays corroborated these findings, showing similar trends (Fig. 3I to L and Fig. S2H to K). Collectively, these results indicated that RBM15 likely functions by regulating the EMT signaling pathway.

### RBM15 mediated the EMT signaling pathway by regulating ECT2

**Fig. 4.**
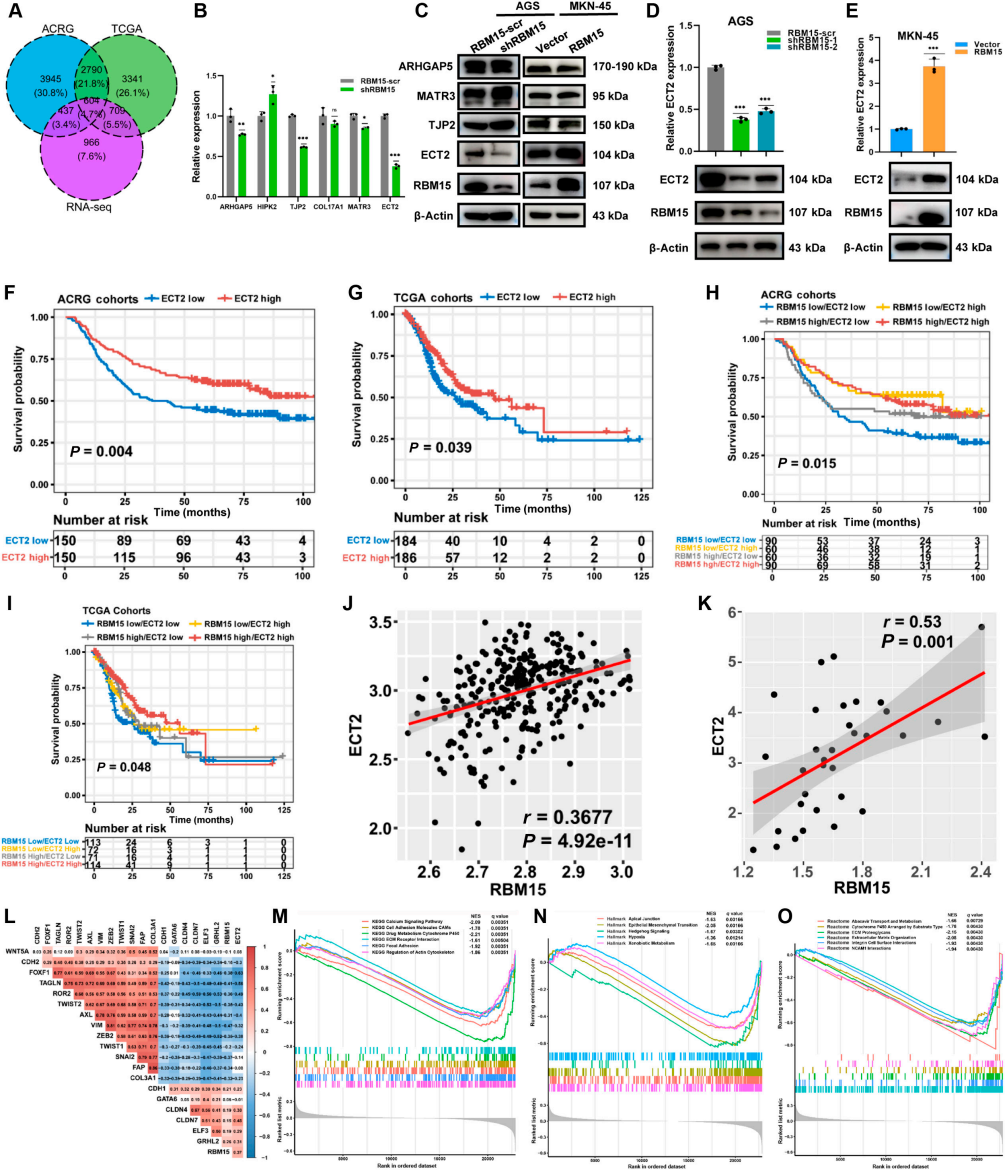
RBM15 mediated the EMT signaling pathway by regulated ECT2. (A) Venn diagram of differential genes of RBM15 in the RNA-seq, TCGA, and ACRG databases. (B) ECT2 expression in RBM15-knockdown AGS cells assessed by qPCR and Western blotting. (C) ECT2 protein levels in AGS and MKN-45 cells after RBM15 knockdown or overexpression. (D and E) ECT2 expression in treated AGS and MKN-45 cells assessed by qPCR and Western blotting. (F and G) Survival analysis of ECT2 in ACRG and TCGA databases [(F): hazard ratio (HR), 0.63; 95% confidence interval (CI), 45 to 0.86; (G): HR, 0.71; 95% CI, 0.51 to 0.98]. (H and I) Joint survival analysis of RBM15 and ECT2 in ACRG and TCGA cohorts [(H): RBM15-high/ECT2-high versus RBM15-low/ECT2-low: HR, 0.51; 95% CI, 0.33 to 0.77; (I): RBM15-high/ECT2-high versus RBM15-low/ECT2-low: HR, 0.56; 95% CI, 0.37 to 0.84]. (J) Correlation between ECT2 and RBM15 in ACRG cohort. (K) Correlation between ECT2 and RBM15 in SDPH cohort. (L) Correlation analysis of RBM15 in ACRG database. (M to O) Enrichment analysis based on the expression of ECT2 in KEGG, Hallmark, and Reactome databases.

The regulatory landscape of RBM15 in GC was mapped through convergent analysis of knockdown-induced DEGs and TCGA/ACRG datasets, identifying 604 target genes including key representatives ECT2, ARHGAP5, HIPK2, TJP2, COL17A1, and MATR3 (Fig. 4A and Fig. S2L to N). Since the expression changes of other molecules, following the interference or overexpression of RBM15, were either inconsistent with predictions or not significant, ECT2 was ultimately selected as the downstream molecule regulated by RBM15. The qPCR and Western blot assays confirmed that ECT2 is a downstream target molecule of RBM15 (Fig. 4B and C). ECT2 expression was reversibly modulated by RBM15 manipulation at transcript and protein levels (Fig. 4D and E). Consistent survival benefits in ECT2-high GC patients (ACRG and TCGA; Fig. 4F and G) prompted investigation of RBM15–ECT2 co-regulation. We therefore implemented quadrant stratification: RBM15-low/ECT2-low, RBM15-low/ECT2-high, RBM15-high/ECT2-low, and RBM15-high/ECT2-high. It was discovered that the RBM15-low/ECT2-low subgroup exhibited the worst prognosis (Fig. 4H and I). Correlation analysis revealed a positive association between RBM15 and ECT2 (Fig. 4J and K). In contrast, RBM15 expression was inversely correlated with key mesenchymal markers, including CDH2 (N-cadherin), VIM, ZEB2, TWIST1/2, and SNAI2 (Fig. 4L). Enrichment analysis based on differential ECT2 expression also highlighted its role in inhibiting the EMT process (Fig. 4M to O). These findings suggest that, like RBM15, ECT2 plays a crucial role in the negative regulation of the EMT process.

**Fig. 5.**
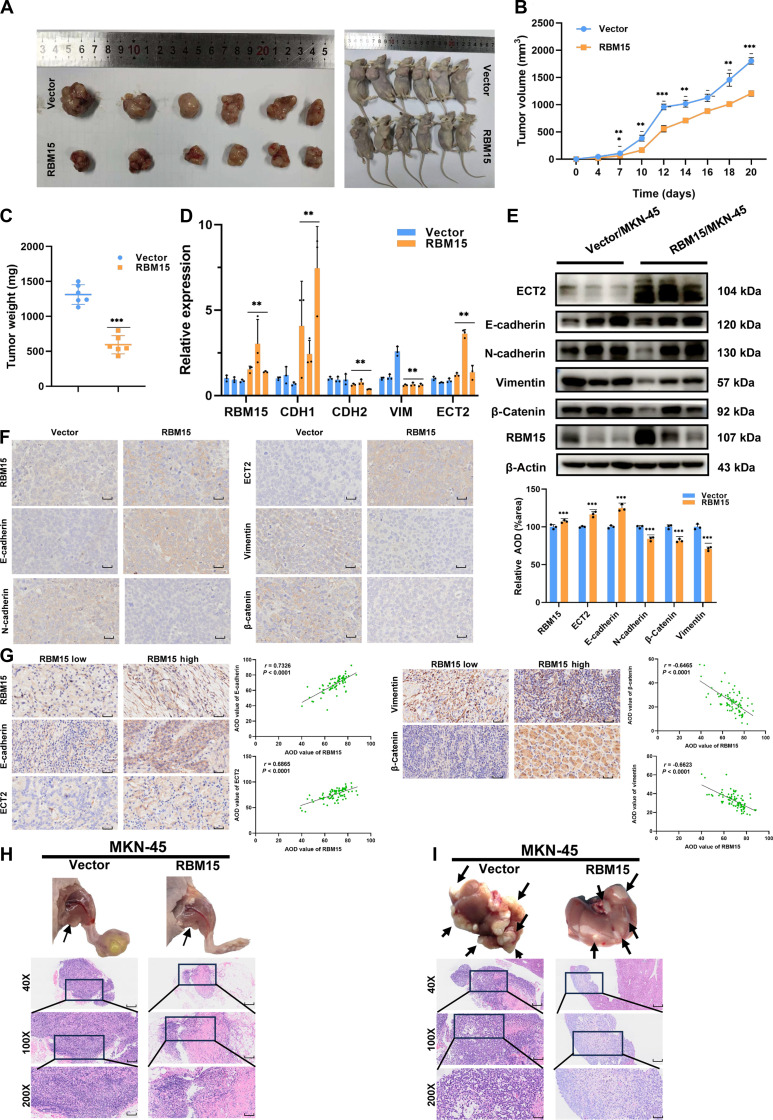
RBM15 inhibits GC progression in vivo by modulating the ECT2-mediated EMT pathway. (A) Representative images of mouse xenograft models and tumors. Upper panel: vector control group (*n* = 6). Lower panel: RBM15 overexpression group (*n* = 6). (B and C) Tumor volume and weight from mice injected with MKN-45 cells (*n* = 6 per group). (D to F) Expression levels of RBM15, ECT2, CDH1, CDH2, and VIM in mouse tumor tissues analyzed by qPCR, Western blotting, and immunohistochemistry (IHC) (scale bar = 50 μm). (G) Representative IHC images showing high or low expression of RBM15, ECT2, E-cadherin, β-catenin, and Vimentin in a tissue microarray containing samples from 29 GC patients who underwent gastrectomy at Shandong Provincial Hospital (Shandong, China) between January 2019 and December 2022 (scale bar = 50 μm). (H and I) Representative images: lymph node and liver metastasis model of GC cells​ with MKN-45 cells of Vector (vector control) or RBM15 (RBM15 overexpression) (40× scale bar = 200 μm, 100× scale bar = 100 μm, 200× scale bar = 50 μm).

To further assess the effects of RBM15 on GC cell progression in vivo, MKN-45 cells overexpressing RBM15 were subcutaneously injected into mice (*n* = 12). Validating experimental hypotheses, RBM15-overexpressing xenografts demonstrated significantly attenuated tumor growth metrics relative to vector controls, with concordant reductions in both volumetric and gravimetric parameters (Fig. 5A to C). Subsequent analysis of RNA and protein levels in the mouse tumors revealed increased expression of CDH1 (E-cadherin) and ECT2 in the RBM15 overexpression group, while levels of CDH2 (N-cadherin), CTNNB1 (β-catenin), and VIM (Vimentin) were decreased (Fig. 5D and E). Immunohistochemical (IHC) validation demonstrated inverse expression patterns: epithelial regulators RBM15, ECT2, and E-cadherin showed intensified staining in RBM15-OE tumors, whereas mesenchymal markers N-cadherin, β-catenin, and Vimentin exhibited attenuated signals relative to vector controls (Fig. 5F). A tissue microarray analysis (*n* = 29) revealed that RBM15-high tumors consistently up-regulated epithelial effectors ECT2 and E-cadherin while suppressing mesenchymal markers β-catenin and Vimentin, corroborating RBM15’s role in epithelial maintenance (Fig. 5G). Due to RBM15 regulating the EMT signaling pathway, we constructed lymph node metastasis and liver metastasis models of GC to further investigate the impact of RBM15 on GC metastasis. Experiments revealed that RBM15 can inhibit lymph node metastasis (Fig. 5H) and liver metastasis (Fig. 5I) of GC.

**Fig. 6.**
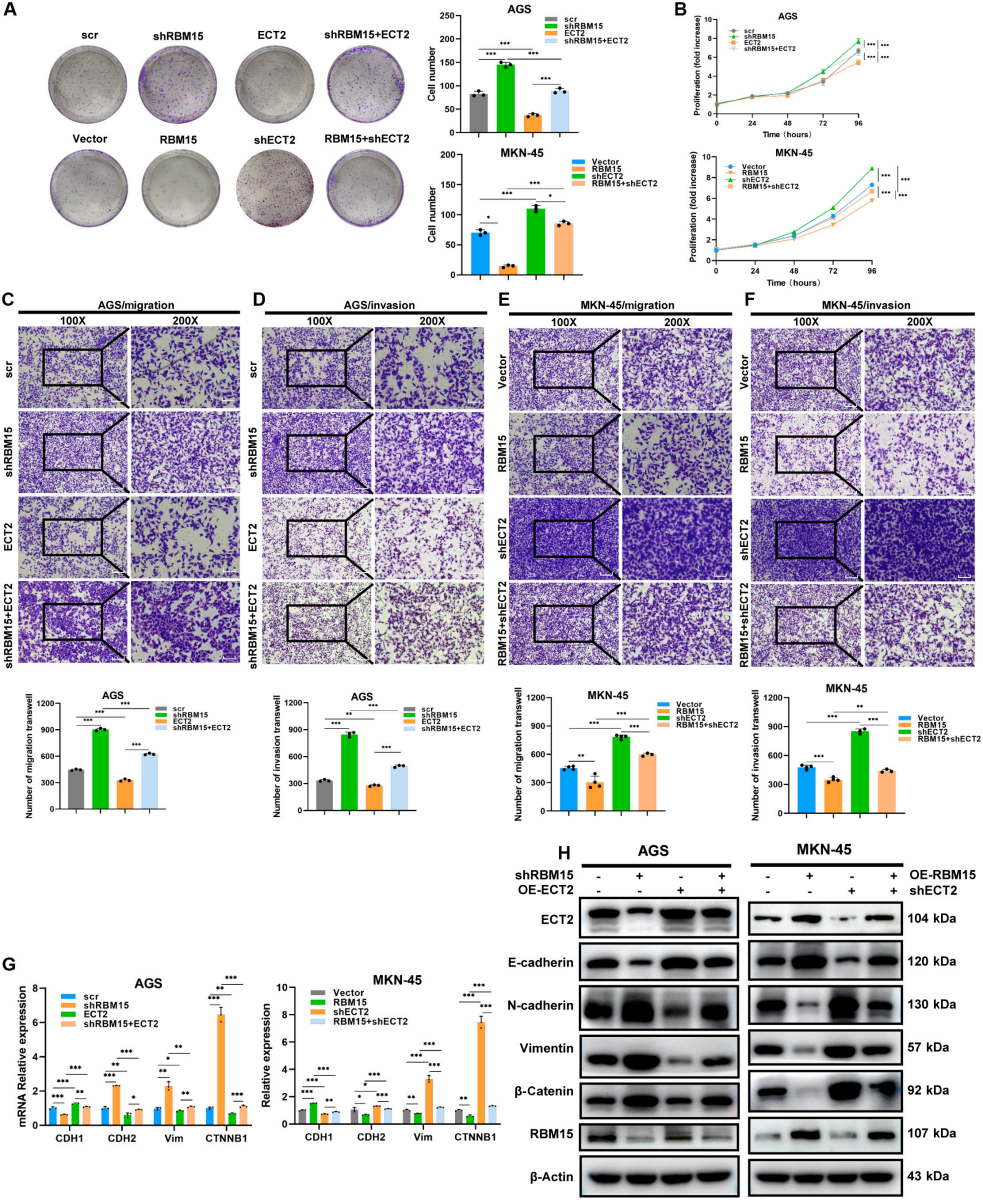
RBM15 inhibits GC cell proliferation and migration by promoting ECT2 expression. (A and B) Cell proliferation assessed by colony formation and CCK-8 assays in AGS and MKN-45 cells after the indicated treatments. (C and D) Migration and invasion assays of AGS cells after the indicated treatments (100× scale bar = 100 μm, 200× scale bar = 50 μm). (E and F) Cell migration and invasion assays in MKN-45 cells after relative treatment (100× scale bar = 100 μm, 200× scale bar = 50 μm). (G and H) mRNA and protein expression levels of CDH1, CDH2, CTNNB1 (β-catenin), VIM, and ECT2 in AGS and MKN-45 cells after the indicated treatments, determined by qPCR and Western blotting, respectively.

Whereas RBM15’s governance of EMT signaling in gastric carcinogenesis was definitively established, the requisite demonstration of ECT2 as the mechanistic conduit remained outstanding. To explore this, AGS cells were transfected with shRBM15 and/or overexpressed ECT2, while MKN-45 cells were transfected with RBM15 overexpression and/or shECT2. These modified cells were subsequently subjected to various functional assays. As shown in Fig. 6A and B, colony formation assay and CCK-8 assays demonstrated that ECT2 overexpression rescued the proliferation ability of GC cells that was diminished by RBM15 knockdown. Conversely, shECT2 reversed the reduction in cell proliferation caused by RBM15 overexpression. Transwell assays further showed that ECT2 overexpression restored the migration and invasion capabilities of GC cells enhanced by RBM15 knockdown, while shECT2 rescued the ability in cells with RBM15 overexpression (Fig. 6C to F). Functional antagonism was observed: ECT2 overexpression rescued RBM15 knockdown-induced mesenchymalization (N-cadherin↑/Vimentin↑/E-cadherin↓), while ECT2 depletion abrogated the EMT-suppressive effects of RBM15 overexpression (Fig. 6G and H).

In summary, RBM15 exerts its regulatory influence on the EMT signaling pathway through the modulation of ECT2, consequently impacting the progression of GC.

### RBM15 mediated methylation of ECT2 and regulated its RNA stability via IGF2BP3

**Fig. 7.**
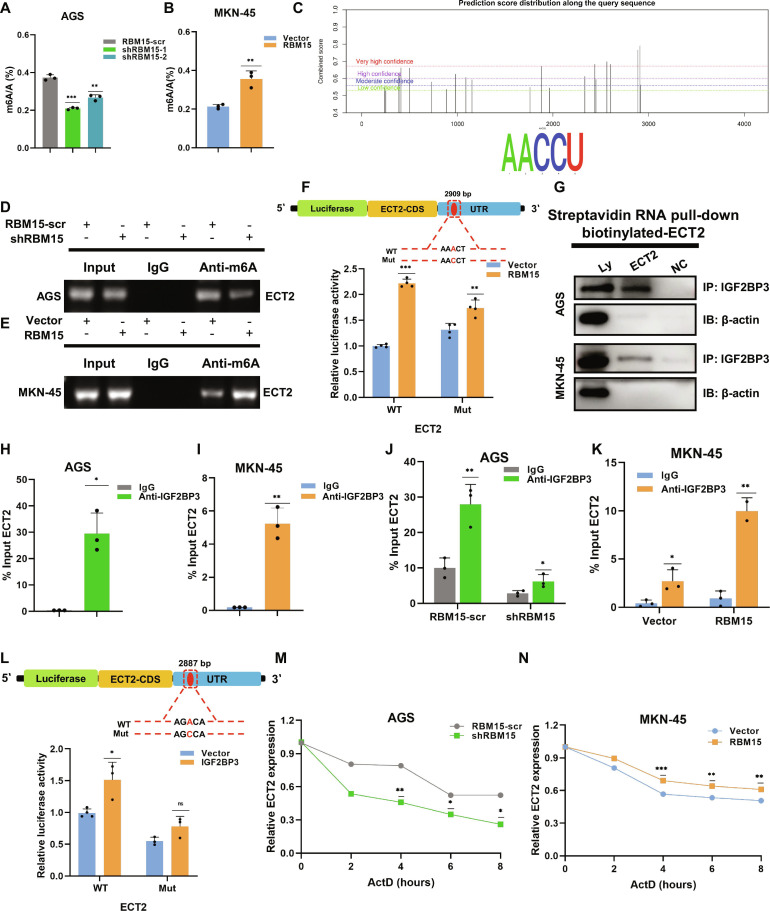
RBM15 regulates m6A-dependent ECT2 modification via IGF2BP3. (A and B) Total methylation level detection in AGS and MKN-45 cells. (C) Prediction score distribution of ECT2 [the bioinformatics tool used for m6A site prediction (SRAMP)]. (D and E) Agarose gel electrophoresis of ECT2 RNA immunoprecipitated by m6A-specific antibody (MeRIP) in AGS and MKN-45 cells. (F) Luciferase reporter assay in 293T cells cotransfected with wild-type (ECT2-WT) or mutant (ECT2-Mut) ECT2 reporter constructs together with vector or RBM15. (G) RNA pull-down assay confirming the interaction between ECT2 and IGF2BP3 in AGS and MKN-45 cells. (H to K) RIP-qPCR was showing the enrichment of IGF2BP3 binding to ECT2 m6A modification sites. (L) Luciferase reporter assay in 293T cells cotransfected with ECT2-WT or ECT2-Mut reporter constructs together with vector or IGF2BP3. (M and N) RNA stability assay showing the half-life of ECT2 mRNA in different transfection groups.

Capitalizing on RBM15’s methyltransferase activity, we deployed the EpiQuik m6A Quantification Kit to measure N6-methyladenosine modifications in RBM15-modulated GC cells, probing its mechanistic control of ECT2. The results showed that m6A methylation levels significantly decreased following RBM15 inhibition and increased with RBM15 overexpression (Fig. 7A and B). Further analysis using the SRAMP methylation prediction website identified potential m6A sites (Fig. 7C). A MeRIP (methylated RNA immunoprecipitation) assay revealed that RBM15 down-regulation reduced the m6A modification of ECT2 mRNA, while RBM15 overexpression enhanced it (Fig. 7D and E). Based on the SRAMP analysis, we mutated the AAACT sequence at 2,909 bp in the 3′UTR to AACCT and conducted a luciferase assay. Overexpression of RBM15 significantly enhanced the luciferase activity driven by the wild-type ECT2 reporter (WT-ECT2). In contrast, while a smaller but statistically significant increase was observed for the mutant reporter (Mut-ECT2), the effect was markedly attenuated compared to the wild-type, demonstrating a mutation-specific regulatory dependency (Fig. 7F).

The regulatory role of “m6A reader” proteins is critical for the functional output of m6A modifications. IGF2BP3 is one such reader that stabilizes target mRNAs, playing a well-documented oncogenic role in various cancers. Its oncofetal expression pattern and involvement in therapy resistance make it a promising diagnostic biomarker and therapeutic target [40]. We examined the enrichment of IGF2BP3 binding to ECT2 mRNA via RNA pull-down assays (Fig. 7G). RIP-qPCR assays confirmed the binding of IGF2BP3 to ECT2 mRNA in AGS and MKN-45 cells (Fig. 7H and I). Moreover, RIP-qPCR assays revealed that RBM15 controls IGF2BP3–ECT2 mRNA binding: Depleting RBM15 impaired complex formation, while overexpressing RBM15 potentiated this molecular interaction in GC cells (Fig. 7J and K). A luciferase assay with a mutated AGACA sequence at 2,887 bp in the 3′UTR to AGCCA further demonstrated that WT-ECT2 luciferase activity significantly increased with IGF2BP3 overexpression, while Mut-ECT2 groups were resistant to IGF2BP3’s effects (Fig. 7L). Additionally, dactinomycin-based transcript stability assays demonstrated RBM15-dependent regulation of ECT2 mRNA: destabilization in shRBM15 cells relative to controls versus stabilization in overexpression models compared to vector groups (Fig. 7M and N).

Synthesizing the evidence, RBM15 emerges as the central regulator that coordinates m6A methylation of ECT2 while enabling IGF2BP3-dependent stabilization of ECT2 transcripts.

### RBM15 enhanced 5-FU sensitivity in GC cells

**Fig. 8.**
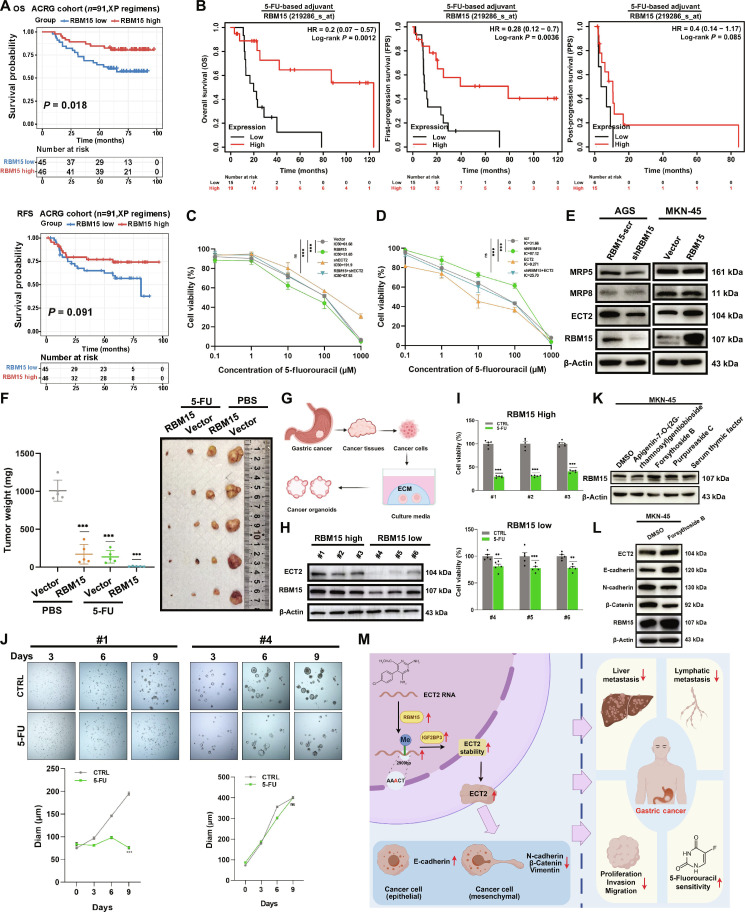
RBM15 enhanced drug sensitivity of 5-FU in GC cells. (A) Survival analysis about OS and RFS (relapse-free survival) with XP chemotherapy in ACRG database. (B) Integrated analysis of GEO datasets evaluating the relationship between RBM15 expression and prognosis (OS, FPS, PPS) in GC patients treated with 5-FU. (C and D) IC_50_ (median inhibitory concentration) values of 5-FU in MKN-45 and AGS cells after the indicated treatments, determined by CCK-8 assay. (E) Protein levels of MRP5 and MRP8 assessed by Western blotting. (F) Images of mouse models and tumors, and tumor weights from mice injected with MKN-45 cells and treated with 5-FU. (G) Schematic diagram of GC organoids. (H) Expression of RBM15 and ECT2 in organoids assessed by Western blotting. (I) Viability of organoids after 5-FU treatment assessed by CCK-8 assay [CTRL: control, 5-FU: 5-fluorouracil (20 μM)]. (J) Diameter of organoids from RBM15-high and RBM15-low groups at different time points after 5-FU treatment [CTRL: control, 5-FU: 5-fluorouracil (20 μM)]. (K) RBM15 protein levels in MKN-45 cells treated with small-molecule compounds (20 μM). (L) Protein levels of ECT2, E-cadherin, N-cadherin, and β-catenin in MKN-45 cells treated with forsythoside B (20 μM). (M) Flowchart of this study.

One of the primary challenges in contemporary GC treatment is the intrinsic or acquired resistance to chemotherapy. This resistance frequently results in unfavorable treatment responses or therapeutic ineffectiveness. In a further effort to explore whether RBM15 modulates drug sensitivity, we delved into the sequencing data of GC patients who underwent 5-FU treatment from the ACRG and TCGA cohorts. The ACRG database encompasses 91 GC patients treated with the XP (Xeloda and Platin) regimen (comprising 5-FU and cisplatin), whereas the TCGA cohort consists of 68 patients who received 5-FU chemotherapy. Improved 5-FU response was associated with RBM15 expression in m6A regulator screening. Furthermore, RBM15 was confirmed to direct m6A methylation on ECT2, with IGF2BP3 interpreting this mark to enhance RNA stability (Fig. S3A). Per Kaplan–Meier analysis, GC patients with high RBM15 expression showed significantly prolonged overall survival and relapse/disease-free intervals when administered XP chemotherapy (Fig. 8A). This indicates that patients with high RBM15 expression may be more sensitive to 5-FU. Multi-cohort GEO validation substantiated this result: Elevated expression significantly correlated with enhanced 5-FU therapeutic response in GC patients (Fig. 8B). In vitro CCK-8 assays demonstrated that shECT2 in MKN-45 rescued the sensitivity of 5-FU that was increased by RBM15 overexpression (Fig. 8C). Overexpressed ECT2 in AGS rescued the sensitivity of 5-FU that was decreased by shRBM15 (Fig. 8D). The Western blot assay found that RBM15 does not affect the multidrug resistance-related proteins associated with 5-FU (MRP5 and MRP8), indicating that RBM15 mediates the drug sensitivity of 5-FU through ECT2 rather than multidrug resistance-related proteins (Fig. 8E). Animal experiments showed that overexpression of RBM15 and injection of 5-FU result in the smallest tumor volume (Fig. 8F).

In order to further visualize the impact of RBM15 on 5-FU chemotherapy and provide value for improving personalized treatments and predicting individual patient response to chemotherapy, we established patient-derived GC organoids (Fig. 8G). Based on RBM15 expression levels in organoid cultures, samples were stratified into high-RBM15 (#1, #2, #3) and low-RBM15 (#4, #5, #6) subgroups. Analysis via Western blotting demonstrated elevated expression of both RBM15 and ECT2 in the high-RBM15 subgroup relative to the low-RBM15 subgroup (Fig. 8H). The CCK-8 assay found that the RBM15-high group exhibited greater sensitivity to 5-FU than the RBM15-low group (Fig. 8I). Furthermore, post-5-FU intervention, the RBM15-low organoids ​​displayed larger diameters,​​ ​​while​​ RBM15-high organoids ​​manifested significant size diminution​​ per neoplastic organoid drug response assays (Fig. 8J and Fig. S3E). Collective evidence established that RBM15 augments 5-FU cytotoxicity in GC cells through ECT2-dependent mechanisms. During the experimental process, it was found that although an experimentally resolved structure exists for human RBM15, we selected the AlphaFold-predicted structure (AlphaFold ID: AF-Q96T37-F1) for virtual screening in HY-L001P (MCE Bioactive Compound Library Plus). This screening aimed to identify small-molecule compounds with binding affinity for RBM15 to enhance RBM15 expression (Table S3 and Fig. S3B). We selected the top 4 small-molecule compounds [purpureaside C, apigenin-7-O-(2G-rhamnosyl)gentiobioside, forsythoside B, and serum thymic factor] and used them (20 μM) to stimulate MKN-45 cells with low RBM15 expression. Subsequently, the expression of RBM15 was detected by Western blot (Fig. 8K). The results showed that forsythoside B increased the expression level of RBM15 and also influenced the expression of the downstream molecule ECT2 and the EMT signaling pathway (Fig. 8L). The results from the colony formation assay and CCK-8 assay demonstrate that the combination of forsythoside B and 5-FU can more effectively inhibit the proliferation of GC cells, exhibiting a certain synergistic effect (Fig. S3D and E). This finding suggests that forsythoside B may target RBM15 as an effective regulator of GC initiation and progression.

## Discussion

Accumulating data confirm​​ that dysregulated m6A methylation ​​participates in​​ tumorigenesis across organ systems, ​​specifically modulating​​ disease progression in acute myeloid leukemia (AML) [41,42], breast cancer [43,44], GC [45,46], lung cancer [47,48], kidney cancer [49], and ovarian cancer [50]. Collectively, m6A-related genes are implicated in complementary mechanisms that drive cancer progression. For example, the methyltransferase METTL3 regulates multiple pathways, such as tumorigenesis [51], development, and metastasis [52]. Specifically, METTL3 modulates the mRNA translation of key oncogenes, including c-MYC [53], BCL-2 [54], and PTEN [55]. Tumor progression is critically driven by m6A-modifying enzymes and their binding partners, as evidenced by breast cancer models where METTL3-IGF2BP3-mediated m6A hypermodification of PD-L1 transcripts impedes immunological surveillance [56]. Similarly, without functional IGF2BP2 recognition​​, METTL3 fails to exert its ​​full protumorigenic effects​​ in colorectal carcinoma [57]. Another key methyltransferase, METTL14, a critical methyltransferase, functions as a tumor suppressor by modulating tumorigenesis, cell migration, and proliferation. Specifically in colorectal cancer, it inhibits metastasis through 2 mechanisms: down-regulation of the oncogenic lncRNA (long noncoding RNA) XIST (X-inactive specific transcript) and mediation of N6-methyladenosine (m6A) modifications on SOX4 mRNA [58,59]. RBM15 is classified as an m6A writer, similar to MELLT3/14, and exhibits methyltransferase activity in the m6A writer complex. There is controversy over whether RBM15 has methyltransferase activity. But according to our findings, RBM15 can regulate the methylation of ECT2 by methyltransferase activity. Tumor progression is modulated by ​​m6A modifier–reader interactions​​, ​​as evidenced in breast cancer where​​ ​​the absence of HNRNPC (heterogeneous nuclear ribonucleoprotein C) binding to VIRMA-generated TFAP2A m6A sites fails to activate DDR1​​. ​​Without this axis​​, ​​disrupted collagen alignment and enhanced immune surveillance occur​​, ​​thereby impeding immune escape [60]. Without KIAA1429-induced m6A hypomodification​​, BTG2 mRNA fails to achieve YTHDF2-mediated stabilization, ​​significantly attenuating tumorigenic potential in lung adenocarcinoma​​ [61]. Investigations into RBM15 remain at a preliminary phase. According to recent reports, RBM15 exhibits ​​oncogenic​​ properties in most cancers (e.g. osteosarcoma [62], lung cancer [63,64], breast cancer [65], and bladder cancer [66]). Targeted depletion of RBM15​​ induces m6A modification of MyD88 mRNA, ​​a key mechanism​​ that ​​significantly reduces​​ proliferative capacity and metastatic potential in colorectal carcinoma cells [25]. In lung cancer, RBM15 down-regulation hindered non-small cell lung cancer (NSCLC) cell proliferation and metastasis by modulating the KLF1-TRIM13/ANXA8 axis [63]. In breast cancer, RBM15 coordinates cancer cell growth through altered serine and glycine metabolism. However, in this study, ​​biological analysis combined with in vitro and in vivo experiments conclusively demonstrates that RBM15​​ acts as a ​​tumor-suppressive​​ factor in GC. Previous studies reported that RBM15 acts as an oncogene in GC [67]. However, those investigations relied merely on expression differences between GC tissues and normal tissues, along with validation in a single knockdown cell line. In contrast, our study comprehensively demonstrates that RBM15 robustly suppresses GC tumorigenesis and progression through multi-database survival analysis (integrating public databases and the in-house SDPH cohort) and functional validation using knockdown and overexpression experiments in GC cells and in vivo models. This study uncovers a critical role for RBM15 in modulating the EMT signaling pathway and the sensitivity of 5-FU through m6A methylation modification of ECT2, mediated by IGF2BP3. RBM15 expression could be leveraged as a clinical biomarker (e.g., IHC scoring in biopsies to guide 5-FU therapy). Current research on ECT2 has been predominantly focused on its role in other tumors. For example, in cervical cancer, ECT2 promotes malignant phenotypes through activation of the AKT/mTOR (mammalian target of rapamycin) pathway and contributes to cisplatin resistance. Research on ECT2 has primarily centered on its functions in other tumors.​​ Specifically in cervical cancer, ​​malignant phenotypes are promoted by ECT2 via AKT/mTOR pathway activation​​, concurrently ​​inducing resistance to cisplatin chemotherapy [68]. The oncogenic role of ECT2 in lung adenocarcinoma involves its governance over ECM homeostasis and integrin-mediated focal adhesion cascades, thereby accelerating tumor advancement [69]. Nevertheless, the functional significance of ECT2 in gastric carcinogenesis ​​has not been fully elucidated​​.

Bioinformatics analysis indicated a positive correlation between elevated RBM15 levels and improved prognosis in GC. Mechanistically, RBM15 was found to regulate ECT2 expression via IGF2BP3-dependent m6A methylation in GC cells. Cellular and in vitro experiments demonstrated that RBM15 inhibits the progression of GC cells. Analyses of public databases and RNA-sequencing (RNA-seq) indicated that RBM15 regulates ECT2 expression. Furthermore, we employed bioinformatics approaches to explore the impact of RBM15 and ECT2 on GC prognosis and validated the relationship between RBM15, ECT2, and the EMT signaling pathways. Rescue experiments confirmed that RBM15 regulates the EMT signaling pathway through ECT2 at the mRNA level. RBM15 binding to the 2,909-bp site on ECT2 governs its methylation and subsequent expression​​, ​​as evidenced by MeRIP-seq (using m6A-specific antibody) and dual-luciferase activity measurements​​. ​​Concurrently, RIP-qPCR and RNA pull-down with biotinylated probes demonstrated IGF2BP3–ECT2 recognition, a process fine-tuned by RBM15. Additionally, analysis of public databases indicated that high RBM15 expression correlates with better outcomes in patients receiving 5-FU chemotherapy. Finally, sensitivity enhancement of GC cells to 5-FU by RBM15 via ECT2​​ was confirmed through in vitro cytotoxicity assays (CCK-8) and patient-derived tumor organoid drug response testing.

## Materials and Methods

### Tissue specimens and patient information

The first cohort (*n* = 84) utilized Shanghai Outdo Biotech TMAs (2011–2012; Fig. 1H), whereas the second cohort (*n* = 29) involved surgically resected GC specimens from Shandong Provincial Hospital (2019–2022; Fig. 5G). Ethics approval (no. 2021-529) and informed consent were obtained, excluding neoadjuvant chemotherapy cases.

### Collect and preprocess of publicly attainable datasets

Retrospective GC gene expression profiles with clinical annotations were curated from open-access datasets: NCBI-GEO, TCGA, Singapore [70], and KUGH [71] cohort. Validation employed 33 tumor-adjacent normal tissue pairs from in-house SDPH cohort [70]. The datasets included the GSE15459 (Singapore), GSE14210, GSE22377, GSE29272, GSE51105, and GSE62254 datasets. RNA sequencing data from TCGA, in FPKM (fragments per kilobase of exon per million mapped reads) format, were downloaded from the UCSC Xena database (https://gdc-hub.s3.us-east-1.amazonaws.com/download/TCGA-STAD.star_fpkm.tsv.gz).

### Gene Set Enrichment Analysis

The 23 m6A genes from prior work [39] informed our GSEA analysis using “clusterProfiler”. This examined pathway differences in RBM15 subgroups against MSigDB v7.1-defined Hallmark, Reactome, and KEGG gene sets [70].

### Cell culture and transfection

MKN-45 cells were obtained from the Key Laboratory for Experimental Teratology of the Ministry of Education, Department of Pathology, School of Basic Medical Sciences, Shandong University, with AGS cells from the American Type Culture Collection. Cells were maintained at 37°C under 5% CO_2_ in RPMI 1640 medium (Gibco) supplemented with 10% fetal bovine serum (PAN Biotech) and 1% penicillin/streptomycin (Thermo Fisher). The cells were plated at 2 × 10^5^ per well in 6-well plates overnight. Lentiviral constructs (Genechem) and small interfering RNA (siRNA) (Keyybio, Shandong, China) were transfected per manufacturer instructions followed by 7-d puromycin selection (0.5 μg/ml, MedChemExpress).

### Quantitative real-time PCR

Total RNA isolation used Trizol (Vazyme), with cDNA synthesis by HiScript III RT SuperMix (Vazyme). Quantitative reverse transcription PCR (qRT-PCR) employed ChamQ SYBR Master Mix (Vazyme) on a QuantStudio 1 system (Applied Biosystems), normalized to glyceraldehyde-3-phosphate dehydrogenase (GAPDH) using Table S4 primers. Relative expression was computed via 2−ΔΔCt (increased values indicate higher transcription).

### Western blot analysis and antibodies

Proteins extracted via lysis buffer/boiling were quantified by BCA (bicinchoninic acid) (Solarbio). After sodium dodecyl sulfate–polyacrylamide gel electrophoresis (SDS-PAGE) (80 V, 2.5 h) and polyvinylidene difluoride (PVDF) transfer (1.5 h), membranes were washed in 1% TBST (tris-buffered saline with Tween 20) (3 × 5 min) and probed overnight at 4 °C with RBM15 (Proteintech 10587-1-AP), ECT2 (Santa Cruz sc-514750), E-cadherin (60335-1-Ig), N-cadherin (66219-1-Ig), Vimentin (60330-1-Ig), β-catenin (66379-1-Ig), IGF2BP3 (14642-1-AP), MRP5 (Affinity DF7149), MRP8 (DF6556), and β-actin (20536-1-AP). Species-matched secondaries (Proteintech SA00001/SA00002) were applied per standard protocol.

### CCK-8 assay

Cells were plated in 96-well plates (5 × 10^3^ per well) 24 h before assay. Proliferation kinetics were quantified using CCK-8 (DojinDo) per the manufacturer’s protocol, with absorbance measured at 0, 24, 48, 72, and 96 h to assess viability and proliferative activity.

### Colony formation assay

After plating at 2 × 10^3^ cells per well in 6-well plates and culturing for 7 d (37 °C, medium refreshed every 4 d), colonies containing >50 cells were quantified by optical microscopy.

### Transwell assay

Transwell inserts (Corning) received 200-μl cell suspensions in serum-free medium: migration assays at 3 × 10^5^ cells/ml (uncoated) and invasion assays at 5 × 10^5^/ml (Matrigel-coated). Lower chambers contained 600 μl of complete medium. Post-incubation, cells were fixed in 4% paraformaldehyde/phosphate-buffered saline (PBS) (30 min) and stained with crystal violet (Sigma-Aldrich, 30 min).

### Wound healing assay

Confluent monolayers (>95%) in 6-well plates were centrally wounded with sterile tips, washed with PBS, and then maintained in 1% serum medium. Wound closure was quantified at 0, 24, and 48 h by measuring edge-to-edge distances in 3 random ×100 fields.

### In vivo experiment

Balb/c male nude mice, 4 to 5 weeks old, weighing 20 to 25 g, were purchased from Vital River Laboratories (Beijing, China). For tumor growth studies, groups of 6 mice each were used to assess the effects of RBM15 overexpression and their respective control vectors. Each mouse was injected subcutaneously with 100 μl of tumor cells transfected with lentivirus (5 × 10^6^). Tumor volumes were measured every 2 d. After 4 weeks, tumors were harvested, measured for volume and weight, and photographed. 5-FU (Selleckchem, USA) was injected intraperitoneally into mice at a dose of 25 mg/kg 3 times a week. Lymph node metastasis model: GC cells were injected into the plantar pad of mice, and the popliteal lymph nodes were harvested after 4 weeks. Liver metastasis model: GC cells were injected into the spleen of mice, and the mice were dissected to harvest their livers 6 weeks later.​ The study was approved by the Clinical Research Ethics Committee (no. 2021-529).

### MeRIP experiment

RNA was isolated using published methods. MeRIP-PCR (EpigenTek P-9018-24) utilized 2 μg of total RNA per the manufacturer’s protocol. Methylated RNA was resolved by 1% agarose gel electrophoresis (100 V, 1 h).

### Luciferase reporter assay

293T cells in 24-well plates were cotransfected with luciferase reporter, Renilla reporter, and RBM15 overexpression plasmid or control vector. Absorbance measurements were completed within 48 h post-transfection.

### RNA stability assays

ECT2 mRNA decay kinetics in RBM15-modified GC cells were quantified via actinomycin D transcriptional arrest (10 μg/ml; HY-17559, MCE). Post-treatment RNA extraction and qPCR analysis adhered to published protocols.

### RIP-qPCR and RNA pull down

For RIP-qPCR: Cell lysis was performed in tris-triton buffer. Lysates were incubated overnight with protein A/G magnetic beads precoated with IGF2BP3 antibodies. Post-washing, bead-bound RNA was quantified by qPCR. In RNA pull-down assays, streptavidin magnetic beads were conjugated with biotinylated ECT2 transcripts. Following 6-h incubation with tris-triton-lysed cell extracts, IGF2BP3 protein binding was detected via Western blot.

### Tumor organoid drug sensitivity test

GC specimens obtained post-gastrectomy (Table S5) were processed through (a) triple rinse in Dulbecco’s PBS/antibiotics, (b) mechanical dissociation into 1-mm^3^ fragments, (c) enzymatic digestion (10 to 15 ml, 37 °C, 30 to 60 min), (d) mesh filtration, and (e) centrifugation (2,000*g*, 4 °C, 5 min). Post-RBC (red blood cell) lysis and recentrifugation, cells were reconstituted in Matrigel (BD; 1:1 v/v) for 24-well plating. PDO (patient-derived organoid) assays utilized 96-well plates seeded with 50 organoid fragments + 10 μl of Matrigel. At 50 μm mean diameter, 10 μM 5-FU (Selleckchem/dimethyl sulfoxide) was administered. Bright-field monitoring at 72-h intervals preceded day 9 viability quantification via CellTiter-Lumi 3D (Beyotime): Post-reagent incubation (100 μl, 5 min vortex, 30 min room temperature), luminescence was measured spectrophotometrically, with diameter analysis performed in ImageJ.

### Mutational landscape profiling and signature analysis

Given that genomic alterations are key determinants of tumor initiation and progression, we profiled the mutational landscape of GC [72]. Subsequent integrative analysis using R’s “maftools” [73] enabled (a) visualization of m6A/SMG mutation spectra via waterfall plots in TCGA-STAD (stomach adenocarcinoma) and (b) extraction of mutational signatures, where Bayesian NMF (non-negative matrix factorization) decomposed mutation portraits into process-defining “signatures” and activity-quantifying “contributions” matrices [16,39].

### Statistical analyses

Statistical workflows employed R v4.0.1 and GraphPad Prism 8. Quantitative variables were stratified by normality: Gaussian distributions used 2-tailed Student’s *t* tests; non-Gaussian distributions used Wilcoxon rank-sum tests. Categorical analyses selected χ^2^ or Fisher’s exact tests per contingency table requirements. Intervariable correlations were evaluated using Spearman’s rank correlation analysis. Associations between RBM15 expression and survival outcomes were analyzed using Kaplan–Meier survival curves and further validated by Cox proportional hazards regression. All experiments were performed with 3 biological replicates, and a two-sided *P* value < 0.05 was considered statistically significant.

## Data Availability

No datasets were generated or analyzed during the current study.
