# Supplementary figures and images for: RBM15 Enhances 5-Fluorouracil Drug Sensitivity and Suppresses Gastric Cancer Progression by Modulating N6-Methyladenosine Modification of ECT2-Dependent IGF2BP3
Source: Research (Wash D C). 2026 Feb 2;9:1108. doi: 10.34133/research.1108 (PMC12862136; doi:10.34133/research.1108)

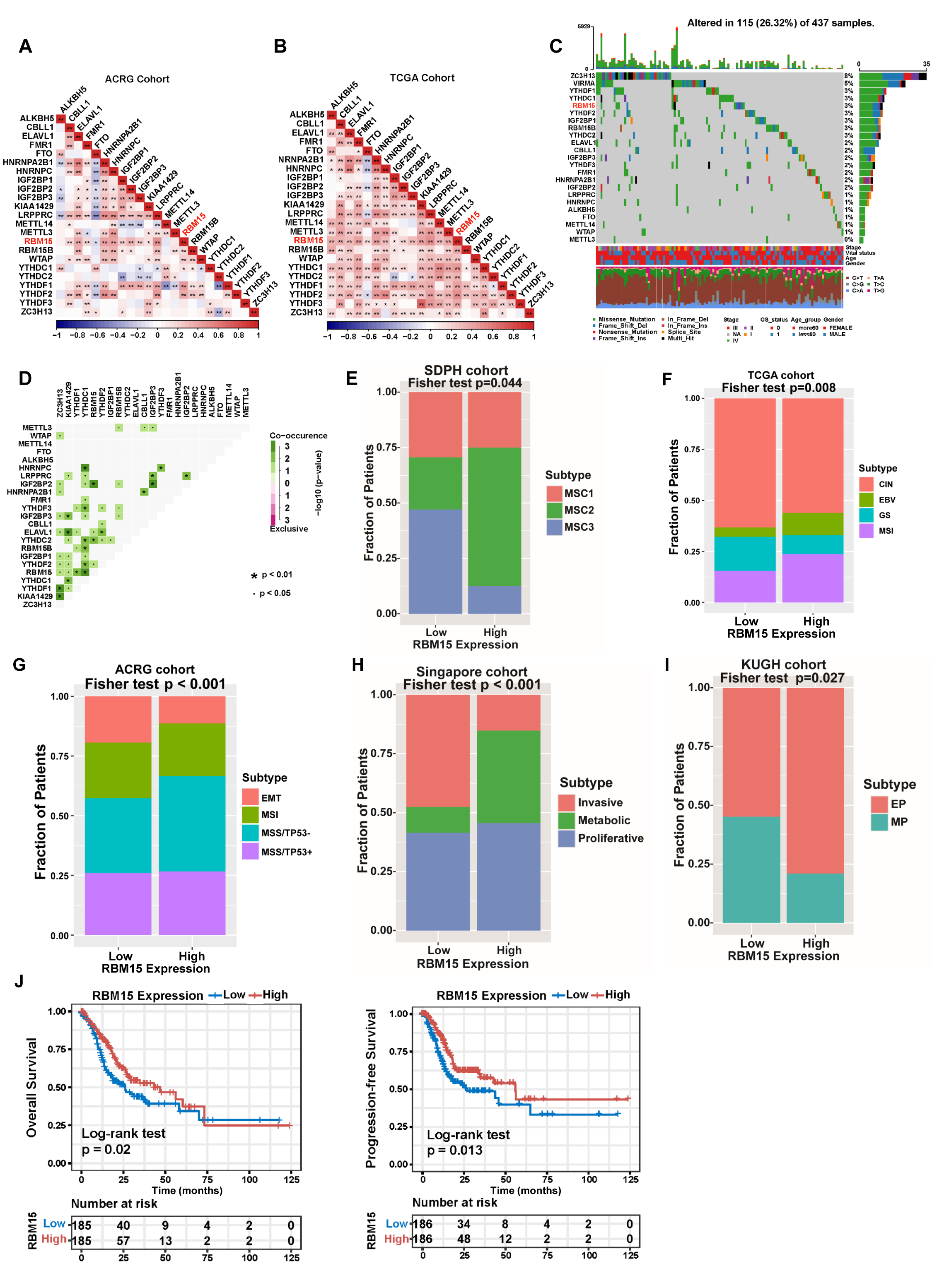

Supplement: Supplementary 1 — Figs. S1 to S3 Tables S1 to S5 [file research.1108.f1.zip › Figure S1.tiff]

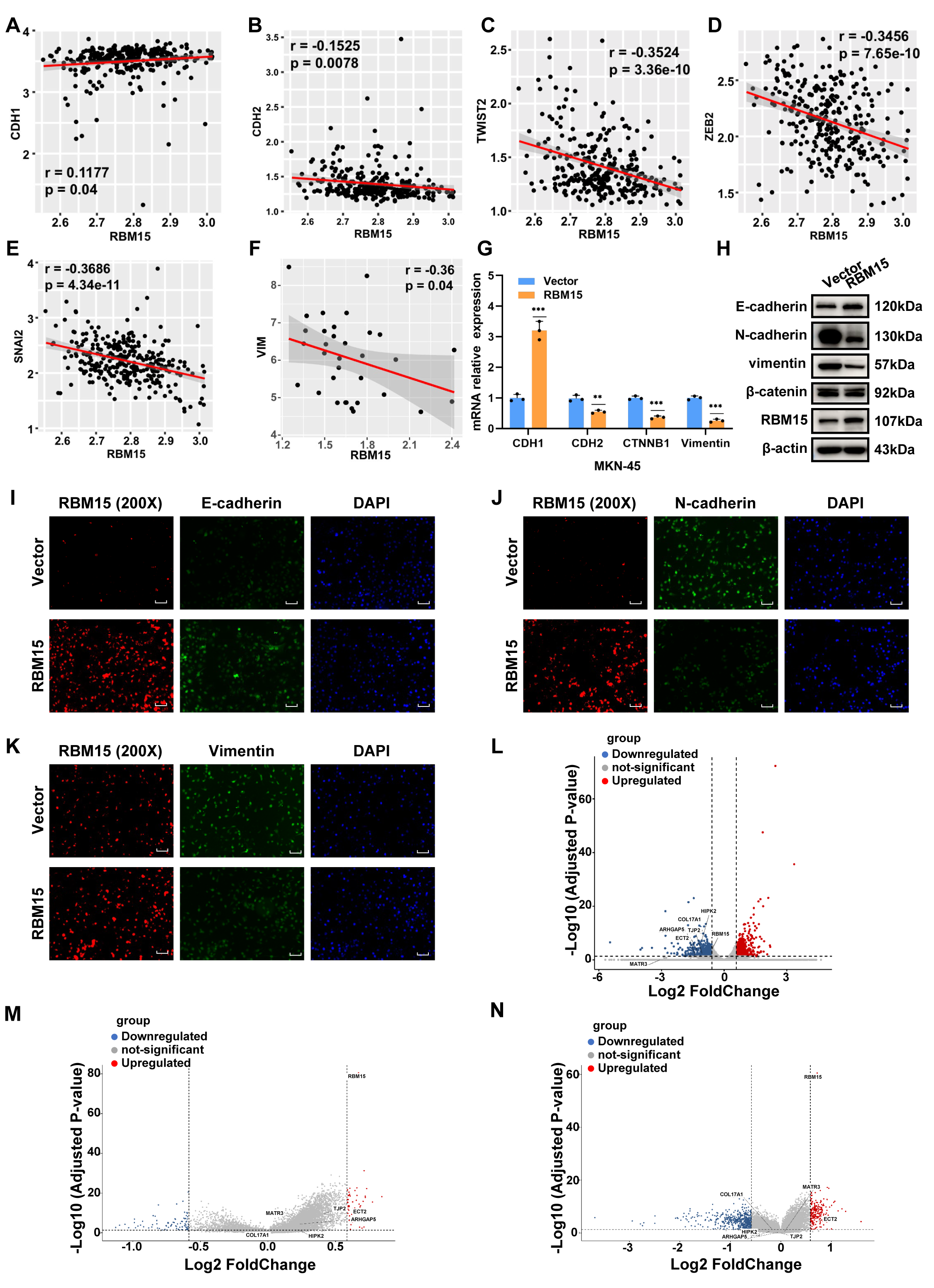

Supplement: Supplementary 1 — Figs. S1 to S3 Tables S1 to S5 [file research.1108.f1.zip › Figure S2.tiff]

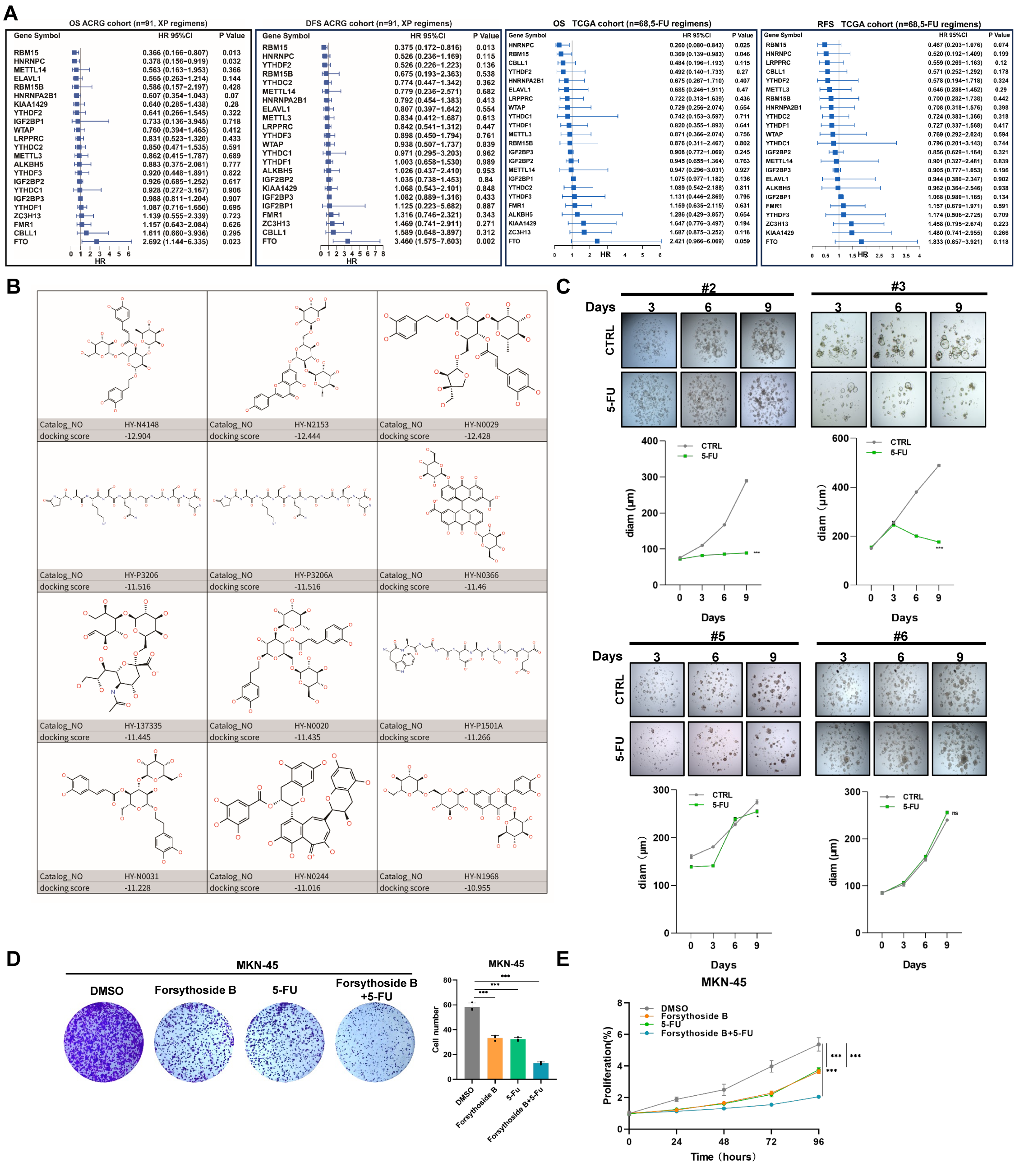

Supplement: Supplementary 1 — Figs. S1 to S3 Tables S1 to S5 [file research.1108.f1.zip › Figure S3.tiff]
